# Qualitative data analysis: reflections, procedures, and some points for consideration

**DOI:** 10.3389/frma.2025.1669578

**Published:** 2025-09-30

**Authors:** Niroj Dahal

**Affiliations:** Kathmandu University School of Education, Lalitpur, Nepal

**Keywords:** qualitative data analysis, intersubjective relations, iterative process, co-construction, theme development, reflexivity, peer debriefing, triangulation

## Abstract

This article adopts a constructivist grounded theory approach based on the principle of intersubjective relations and the co-construction of interpretations. Reflecting on the author's experiences as a tutor, supervisor, examiner, and reviewer to demystify qualitative data analysis (QDA), this article emphasizes member checking with participants to confirm the researchers' interpretations and collaboratively constructed meanings, addressing reflexivity, peer debriefing, and triangulation. QDA is framed as an iterative, dynamic process of extracting meaning from diverse data forms (field text, narrative, voice, reflective note, text, audio, and video). The procedures of data analysis and/or writing as a process of inquiry, such as data immersion, initial impressions, codes, categories, and theme developments, are explored across methods or methodologies, including autoethnography, participatory action research (PAR), narrative inquiry, grounded theory, phenomenology, ethnography, case study, and other alternative research methods or methodologies, with data saturation as the final stepping point when no new data and/or insights from field text, narrative, voice, reflective note, text, audio, or video are extracted. Challenges like generating an intersubjective construction of meaning with research participants and achieving data saturation are addressed through methods such as reflexivity, peer debriefing, member checking and triangulation. This article provides a practical guide for scholars on data analysis, incorporating reflections, procedures, and some points for consideration to ensure rigor and meaningful analysis.

## 1 Introduction

This article explores and documents the author's journey and experiences in learning and applying qualitative data analysis. This journey encompasses mastering both deductive and inductive approaches, alongside specific methods including content analysis, thematic analysis, narrative analysis, discourse analysis, and phenomenological research. The exploration spans from the initial stages of learning these methods through to achieving saturation across diverse qualitative methodologies: autoethnography, participatory action research (PAR), narrative inquiry, grounded theory, phenomenology, ethnography, and case study. In autoethnography, data analysis is considered writing as a process of inquiry that involves reflexively interpreting personal experiences (the auto) to illuminate broader cultural or political understandings (the ethno), weaving the self into the social. In PAR, data analysis is a collaborative, cyclical process of collective reflection on data with co-researchers to produce knowledge that directly informs and empowers further action for social change. Data in narrative inquiry is analyzed by restorying lived experiences into a coherent narrative framework, focusing on elements like plot, setting, characters, and themes to uncover the meaning individuals ascribe to their stories. In grounded theory, data are analyzed through iterative cycles of coding (open, axial, selective) to fracture and compare data, forcing the emergence of conceptual categories that are integrated into a core theoretical framework explaining a process. Data analysis in phenomenology involves systematic reflection on rich experiential descriptions to identify and distill the essential, invariant structures (the essence) of a shared lived experience. In ethnography, data are analyzed through immersion and iterative interpretation of cultural patterns, behaviors, and artifacts to develop a holistic, thick description of a shared culture or social group. And, in a case study, data analysis involves a holistic, in-depth examination of a bounded system (the case) to identify patterns and themes that provide insight into the complexity of that particular case within its real-life context.

Qualitative research comprises various methodologies, each necessitating a unique data analysis approach (Naeem et al., 2023) and co-constructing analyses with participants. Qualitative data analysis itself employs deduction and/or induction approaches, offering distinct pathways to understanding data. With deductive analysis, a previous theory is used to analyze and understand the collected data (Pearse, 2019), while inductive analysis makes it possible to determine patterns and insights that can be found in the data (Azungah, 2018), taking care of the intersubjective construction of interpretations/analyses. Though Charmaz (2009), in breaking with the implication that researchers must rely solely on deduction or induction, championed abduction—an imaginative, inferential leap—as a vital tool for grounded theorists to see how social structures are reproduced and potentially altered in service of a social justice agenda while analyzing the data. Thus, abductive inference can also be used in data analysis to offer a way of seeing things that can help researchers and participants appreciate how social structures are reproduced. Content analysis broadly applies to the process that systematically breaks up a dataset into pieces and then attempts to summarize and tabulate the information (Hsieh and Shannon, 2005; Kohlbacher, 2006). However, checklists shall be used for member checking. This approach is where “data, analytic categories, interpretations, and conclusions are tested with members of those stakeholding groups from whom the data were originally collected.” (Lincoln and Guba, 1985, p. 314). Lincoln and Guba (2013) make the point that member checking is a process that involves participants in co-constructing interpretations, analytic categories, and insights. So, member checking also involves checking analytic categories by discussing these with stakeholder groups as part of the process of arriving at conclusions/insights. Thematic analysis is an approach of analysis and reporting that focuses on identifying and analyzing patterns (themes) within data (Braun and Clarke, 2006; Castleberry and Nolen, 2018; Majumdar, 2022; Nowell et al., 2017). However, these themes are not “in” the data but are based on inferences that go beyond “the data” that can be checked in discussion with participants/stakeholders so that they can be modified, adjusted, or added to (to name but a few). Narrative analysis is defined as examining the stories people tell themselves to make sense of their experiences (Cowger and Tritz, 2019; Riessman, 1993). The grounded theory can contribute to revealing issues that tend to be the cause of phenomena of interest areas (Byrne, 2001; Corbin and Strauss, 1990; de Moura et al., 2022; Glaser and Strauss, 2017). Thus, data analysis in grounded theory is an iterative process of initial and focused coding, constant comparative analysis, and theoretical memo-writing to construct a theory that emerges from the data and is co-created through the researcher's interaction with participants (Charmaz, 2008). Discourse analysis interrogates how language is used in specific social and contextual settings (Aydin-Düzgit and Rumelili, 2019; Bonyadi, 2019), and interpretative phenomenological analysis (IPA) examines participants' understanding and meaning-making of their personal lived experience (Pringle et al., 2011; Smith, 2011; Smith and Osborn, 2008; Tuffour, 2017). These various techniques collectively enable a rich understanding of qualitative data while addressing reflexivity, which for many qualitative researchers involves being responsive to participants' and stakeholders' suggestions on how to develop a “rich” understanding in a co-constructed process.

Each methodology also dictates specific analytical lenses. Autoethnography connects deeply personal experiences to broader cultural, political, and social meanings, using the researcher's own life as primary data (Dahal and Luitel, 2022; Ellis et al., 2010; Holdsworth, 2024; Luitel and Dahal, 2021). PAR engages community members as partners from start to finish in the research process related to the issues they are dealing with directly and involves cooperation, action, and social transformation (Baum et al., 2006; Cornish et al., 2023; Kidd and Kral, 2005; Kindon et al., 2007). Narrative research collects and analyzes personal stories, autobiographies, diaries, chats, and narratives that are told or written to discover how people understand their lives and experiences (Connelly and Clandinin, 1990, 2012; Mertova and Webster, 2019; Wei, 2023). Grounded theory is a method of systematically gathering and analyzing data, frequently through coding and constant comparison, until theory saturation is achieved (Barsalou, 2008; Bowen, 2009; Charmaz and Thornberg, 2021; Chun Tie et al., 2019; Corbin and Strauss, 1990). Phenomenology explores the essence of human experience. Often conducted using in-depth interviews and themetic analysis (Neubauer et al., 2019; Reed, 2023). Ethnography uses close observation and context-sensitive field notes to capture cultural events (Herbert, 2000; Soundy and Heneghan, 2022). Case study investigates particular cases (people, groups, events) in their real-life contexts and offers rich qualitative data on intricate phenomena (Baxter and Jack, 2008; Merriam, 2009). So, the researcher's and the examiner's experiences reveal that the data analysis process is essentially a feedback and reflexive loop process. It moves from becoming acquainted with the data to getting deeper into it (Nassaji, 2015; Stuckey, 2015; Thorne, 2000). This involves initial set-up and engagement with the data by reading transcripts, field notes, or playback of recordings (Hansen et al., 2025). Next steps consist of recognizing initial patterns, coding data, and then jotting down preliminary thoughts (Galanis, 2018), thus preparing the ground for generating themes by clustering codes into broader categories and uncovering patterns in the data (Belotto, 2018; Saldaña, 2013, 2016, 2021). However, saturation is the critical endpoint at which new data no longer yield new meanings, insights, or themes, indicating data saturation and thematic sufficiency (Fusch and Ness, 2015; Naeem et al., 2024). Throughout this pursuit, issues such as bias on the part of researchers, volume of data, and the need for rigor are essential (André, 2020; Benedict et al., 2024; Belotto, 2018). To counter these challenges, peer debriefing, member checking, triangulation, and retaining reflexivity are important strategies to be employed to ensure rigor and meaningful analysis (Nobel and Joanna, 2015).

Finally, analysis of qualitative data seeks to interpret nonnumeric data to identify patterns, themes, and perspectives in an unstructured or unfolding data set. Data saturation is identified when no new data point is presented (Rahimi and Khatooni, 2024), and it is important to establish the breadth and credibility of findings and/or insights. The main steps of this analysis are summarized in Table 2. While there is a rich plethora of qualitative methods and analytical techniques, they share the same aim: to attain saturation, a point of diminishing returns, in which the addition of further data does not reveal any further categories or themes that contribute to the depth and tightness of the study (Blandford et al., 2016; Dahal, 2023). Therefore, appreciating and implementing saturation within the context of individual methods and analyses is crucial for qualitative researchers to conduct strong, valid studies. The iterative and inductive approach characteristic of qualitative research is especially fitting for learning about the complexity of human experience and social processes (Aspers and Corte, 2019; Lim, 2024). A key aspect of this journey—that starts with the encoding of the first data and ends with saturation—is crucial, particularly for novice researchers, to ensure the robustness, the depth, and the trustworthiness of the findings or insights (Dahal, 2023; Naeem et al., 2024).

## 2 Constructivist approach as a theoretical referent

This article is based on constructivist epistemology and formulates constructivism as its core theoretical reference point. The constructivist perspective believes that knowledge and meaning are not an absolute truth that already exists but are constructed in the human mind, individually as well as collectively, through human interaction and experience in a social, cultural, and historical background (Dahal and Luitel, 2022; Ellis et al., 2010). QDA is deconstructed and interpreted based on this theoretical position, which, in turn, informs the author's writing. A fundamental aspect of this position is the refutation of a single, verifiable truth and the assertion that reality is multiple and co-constructed. This directly touches upon the presented QDA procedure, which means that the meaning is not passively “extracted” right out of the data but is actively constructed by the researcher by encountering it constantly. One's interpretation arises from the researcher's encounter with the data in the context of the particular data and the heuristic perspective. For that reason, in constructivism, the researcher is not seen as a neutral listener but as the principal tool for collecting and analyzing information. The use of autoethnography by the author—explicitly drawing on the role as a tutor, a supervisor, an examiner, and a reviewer—enrolled this principal (Ellis et al., 2010; Holdsworth, 2024; Luitel and Dahal, 2021). The researcher's reflexivity, background, biases, and positionality are thus not problems to be eradicated but are the meaning-making work; they constitute the data and actively shape analysis. This calls for a deep reflexivity as a fundamental practice, which is overly underlined in the article as important for researchers to engage in, as self-reflectively as possible, on their own assumptions, preconceptions, values, and power (Dahal and Luitel, 2022; Dahal, 2023).

The documentation of reflexive engagement in the researcher's diary exemplifies this commitment to controlling for bias and increasing rigor. The constructionist perspective is thus entirely consistent with the portrayal of QDA as iterative and fluid. Analysis is iterative—not linear, cycling back and forth between immersion in the data, coding, categorization, theme development, and abstraction (Nassaji, 2015; Stuckey, 2015). The discussion process takes place over time, and insights will unfold slowly to reveal understanding between the researcher and the data later. This sense of recursion supports the transition from initial acquaintance to saturation, a phenomenon shaped by the constructivist epistemology. Saturation, at which new data do not offer any new piece of information, is a relative and not an objective standpoint, but this is an endpoint constructed by the researcher through logical and reflective analysis within the given conditions and the scope of exploration (Fusch and Ness, 2015; Naeem et al., 2024; Rahimi and Khatooni, 2024). It indicates that the researcher fully grasped the interpretations constructed with respect to the research questions. Techniques that were recommended to achieve rigor—peer debriefing, triangulation, and member checking—are also congruent with constructivist assumptions. They do not go for complete objectivity but only in order to enhance the credibility, dependability, and confirmability of the constructed interpretations (Dahal, 2023; Morgan and Nica, 2020; Nobel and Joanna, 2015). Peer debriefing, subjective interpretations to scrutiny by those who know the context, triangulation looks for convergence of meaning from various sources or perspectives, and member checking seeks to confirm interpretations with participants, recognizing them as constructors of meaning. Focusing on the constructivist referent offers the philosophical underpinning to seek QDA not as a mechanical method for extrapolating pre-existent truths but as a live, interpretative, and profound personal process of making meaning. Drawing on this interpretation, the author positions the experiences and practical guide, focusing on the active role researchers play, the requirement to be reflexive, the non-linear process of analysis, and experience-based saturation, as topological signposts in nuts and bolts and strictly qualitative research that abides by constructivist premises.

## 3 Method

This study is grounded in a constructivist worldview (Charmaz, 2008) and employs autoethnography, using the researcher's experiences as the primary data sources. This approach aims to clarify the qualitative processes involved in data analysis across various methodologies, such as autoethnography, participatory action research (PAR), narrative inquiry, grounded theory, phenomenology, ethnography, case study, and other alternative research methods or methodologies (Ellis et al., 2010; Holdsworth, 2024; Luitel and Dahal, 2021). Autoethnography facilitates critical self-reflection by the author, who serves as both researcher and practitioner (Dahal and Luitel, 2022). The analysis includes a review of the author's teaching practices as a course tutor, supervisor, external examiner, and reviewer.

### 3.1 Data collection

Data were harvested from the researcher's personal journal kept during doctoral study (February 2020–February 2025), with scrutiny of extracts from 2023–2024 in QDA processes. Submissions reflected reflexive engagement in relation to QDA across methodologies of QDA, including autoethnography, PAR (Participatory Action Research), narrative inquiry, grounded theory, phenomenology, ethnography, and case studies (Dahal et al., 2024). Analytical methods included deductive/inductive analysis, content analysis, thematic/narrative analysis, discourse analysis, and phenomenological analysis (Braun and Clarke, 2006; Riessman, 1993; Smith and Osborn, 2008).

### 3.2 Data analysis procedure

Following an iterative design (Nassaji, 2015; Stuckey, 2015), the analysis started by reading the diaries repeatedly to become more familiar with the diary data (Hansen et al., 2025). We began by coding for descriptive labels of major stages of QDA learning/application (Galanis, 2018), and then engaged in memo-writing to reflect on and initial findings (Saldaña, 2013). For theme development, the codes were categorized, synthesized into higher-order themes, and applied across the data through constant comparison (Belotto, 2018; Saldaña, 2016, 2021). Saturation was the point where no new themes were generated from the data to inform the analysis (Fusch and Ness, 2015; Naeem et al., 2024).

### 3.3 Rigor and trustworthiness

Several methods to establish the analytical credibility were used: peer debriefing—colleagues at Kathmandu University School of Education reviewed quality themes (Morgan and Nica, 2020); triangulation—the cross-validation of diary data with supervisory/examiner experiences (Nobel and Joanna, 2015); reflexivity—whereby the researchers reflected critically on the positionality and their biases in the course of the analysis (Dahal and Luitel, 2022); and member checking—the preliminary findings were presented to peers for feedback given (Dahal, 2023).

### 3.4 Ethical considerations

Ethical approval was obtained from Kathmandu University, School of Education, Lalitpur, Nepal (Ref: PhD-2020febNDRP012; February 20, 2023). Anonymity was achieved by removing identifiers from the diary excerpts.

## 4 Analysis

### 4.1 Beginning the analysis: early steps

The qualitative data analysis process typically commences as data collection unfolds, enabling researchers to shape questions, delve into emergent issues, and discern patterns (Tomaszewski et al., 2020). First, I saturated myself with the various manifestations of the data by reading and rereading transcripts, field notes, or other qualitative data I had collected, making some initial impressions and observations in my journal to allow for further analysis (Stuckey, 2015). Analysis follows, including quotes and coding (where data sections are tagged with interpretative or descriptive labels, which can be inductive, driven by the data, or deductive, taking pre-existing theories or frameworks). Similar codes are then categorized into codes and themes to make an intellectual sense of the data, which often involves the construction of a coding frame or thematic atlas (Ravindran, 2019). Thus, the preliminary stage of data analysis in a qualitative study is crucial for laying the foundation for a robust and meaningful investigation. This stage helps shape researchers' understanding of the study's purpose and/or objectives and guides the overall analysis process.

So, it begins with organizing and getting to know the data (Wardah, 2021), which means I read and reread what I have of the transcripts, field notes, and datasheet to make sense of it. Therefore, first-cycle coding refers to recognizing salient phrases, sentences, or passages in relation to the research question, and often this coding is descriptive in nature to make sense of the data coherently. I also wrote the memo for more context. Memoing is key to this activity, as it allows for thoughts, interpretations, and more general themes to be captured and reflected on to generate insight. I used the initial codes to construct a more organized coding frame to support further analysis now and to encourage and maintain coherence in later stages of analysis. Through continuous development of code and category lists (and higher-order concepts or categories in Table 2), generative analysis produces an eventual data analysis tool. Early engagement with the existing literature helps to orient findings/outcomes and results and determine the gaps the research work can fill. These initial stages are crucial to developing a rigorous approach to analysis, which results in valuable and credible qualitative research findings and/or products. Table 1 above represents the early analysis process.

**Table 1:** Early-stage data analysis workflow.

**Stage**	**Actions**	**Purpose**
Immersion	Reading and rereading data materials.	To gain familiarity and identify patterns.
Initial impressions	Noting observations and first impressions.	To guide further analysis and questions.
Coding	Assigning labels to significant data chunks.	To organize data for deeper analysis.
Grouping codes	Organizing codes into broader categories or themes.	To identify patterns and relationships.
Memo writing	Writing reflections and capturing interpretations.	To document insights and refine ideas.
Framework development	Generating themes.	To guide consistent, thorough analysis.

Source: Personal Diary, August 2023.

### 4.2 Developing deeper insights: moving toward themes

As coding continues (see Table 2), I am able to chart patterns, relationships, and themes in the data (ATLAS.ti., n.d.). The second part of the analysis is the thematic part, consisting of constructing themes by clustering hierarchically related codes and their relations (see Table 2). I distill the theme to the pure essence of the data. The analytic approach of constant comparative analysis allows for systematic comparisons of data segments, codes, and categories to check for consistency and contradictions and keep the analysis grounded in the data. Analytic memos also summarize my thoughts, revelations, and conclusions—both inductive and deductive—as the data analysis proceeds, to follow the progression of the themes and preserve an audit trail of the analysis. In the qualitative data analysis process, the focus moves beyond the description of the data, called initial coding, to gaining a deeper understanding and a more illuminating, nuanced development of the theme(s) (Naeem et al., 2023). This entails systematically reading through the first codes and categories to identify patterns, similarities, and differences that can assist in forming the codes into more global themes. These similar codes are then clumped together thematically, and data are grouped into more abstract and meaningful categories. All themes are well elaborated to represent various co-related codes that are completely defined and distinct.

**Table 2:** From the quotes to the theme.

**Quotes**	**Codes**	**Categories**	**Theme**
• There never seems to be enough time in the day. Often, I find myself too exhausted to focus on reading • Time constraints from work and professional growth are significant • Strain my time • Consequently, time is a constraint in both my personal and professional life.	Time pressures	Time constraints and exhaustion	Barriers to reaching learning goals
• Day-to-day work and professional development present more pressing issues.	Tired		
• Numerous pressing matters, such as virtual meetings, overly ambitious faculty engagements, and assisting students with their learning	Professional development/ support system	Competing demands	
• At home, I want to engage in other activities, but fatigue from the office limits my productivity. • The primary challenge lies in balancing work and family responsibilities.	Balancing life and work		

Source: Personal Diary, December 2023.

Hence, I inferred that thematic mapping depicts the themes and their relationships and enables researchers like me to comprehend how certain themes are linked to subthemes. The iterative refinement process entails revisiting the data and themes, adjusting them to reflect the data accurately (Morgan and Nica, 2020). I subsequently recognized that incorporating these themes with the current theories and literature locates the findings within the wider field and advances the theoretical contribution of/for the study. Lastly, validation consists of gathering peer or participant feedback to verify the reliability and credibility of the analysis (Dahal, 2023; Morgan and Nica, 2020). To improve the trustworthiness and credibility of the analysis, I used techniques including member checking, peer debriefing, and triangulation with colleagues at Kathmandu University School of Education, Nepal. They noted that the Table 2 findings are appropriate and well-articulated, and were able to sufficiently summarize what was represented in the table to some extent. Working toward themes allows for a more in-depth understanding of the data (Braun and Clarke, 2022), making it essential to identify the deeper meanings and patterns concerning the research question.

### 4.3 Approaches to data analysis, goals, data types, and research questions

Starting with autoethnography, which aims to connect personal experiences to broader cultural, political, or social contexts by using personal reflections, journals, and autobiographies (Dahal and Luitel, 2022; Ellis et al., 2010; Holdsworth, 2024; Luitel and Dahal, 2021). The research question focuses on how personal experiences reflect and contribute to understanding a larger cultural or social phenomenon. Participatory Action Research (PAR) aims for change through working with participants and tackling real-world issues using data from observations, focus groups, interviews, and feedback from participants (Baum et al., 2006; Cornish et al., 2023; Kidd and Kral, 2005; Kindon et al., 2007). The central question addressed by PAR is how the research process can be used to empower participants and resolve the problem or issue. Narrative inquiry examines and understands experiences through storytelling, collecting data from stories, interviews, and personal accounts (Connelly and Clandinin, 1990, 2012; Wei, 2023). It aims to uncover insights by examining participants‘ stories about their experiences. Grounded theory is designed to develop a new theory grounded in the data collected, which includes interviews, observations, and documents (Barsalou, 2008; Bowen, 2009; Charmaz and Thornberg, 2021; Chun Tie et al., 2019; Corbin and Strauss, 1990). It is the question of what theory or construct can be extracted from empirical data and analysis in a systematic manner. Phenomenology takes an individual's subjective lived experience as a phenomenon of interest and uses first-person narratives, interviews, and reflections to explore and describe the essence of the experience (Neubauer et al., 2019; Reed, 2023). It seeks to answer the question: What is the essence of participants' lived experiences regarding a specific phenomenon? Ethnography studies and understands cultural groups and practices in their natural settings. Drawing on field notes, participant observation, and interviews, it examines how cultural practices and group dynamics inform behavior and interactions in a particular community (Herbert, 2000; Soundy and Heneghan, 2022). Ethnography addresses the question: how do cultural practices and group dynamics shape behavior and interactions within a specific community? Lastly, case studies investigate a specific instance, organization, or event in depth. It uses interviews, documents, and observations to learn from an in-depth analysis of the particular case or situation (Baxter and Jack, 2008; Merriam, 2009). Case studies aim to answer the question: what can be learned from an in-depth analysis of this particular case or situation?

Likewise, deductive analysis tests existing theories or hypotheses using structured data, coded data, and theoretical frameworks (Pearse, 2019). The research question focuses on how the data supports or contradicts the existing theory or hypothesis. Inductive analysis seeks to generate new theories or patterns from raw qualitative data, such as interviews or observations, by identifying patterns or themes that emerge without pre-existing frameworks (Azungah, 2018). Content analysis quantifies and analyzes the presence of certain words, phrases, or concepts within textual data, including documents, transcripts, or media, to determine frequently occurring concepts, words, or patterns (Hsieh and Shannon, 2005; Kohlbacher, 2006). The thematic analysis identifies and analyzes patterns or themes within qualitative data, such as interviews or focus groups, to uncover recurring themes or patterns that represent the meaning of data (Braun and Clarke, 2006; Castleberry and Nolen, 2018; Majumdar, 2022; Nowell et al., 2017). Narrative analysis aims to understand how individuals construct meaning through their stories or experiences by examining stories, personal accounts, and biographies (Cowger and Tritz, 2019; Riessman, 1993). In discourse analysis, language use is examined in its social and cultural contexts: spoken and written communication and media texts are engaged with to understand how language mirrors or configures social, cultural, or power dynamics (Aydin-Düzgit and Rumelili, 2019; Bonyadi, 2019). Phenomenological inquiry examines and explicates participants‘ lived experiences in their own words (Pringle et al., 2011; Smith and Osborn, 2008), as manifest or as gleaned from their testimonies, reports, and reflections in or on the essence of participants' lived experiences about a particular phenomenon (Smith, 2011; Tuffour, 2017). Arriving at this stage, I can remark that coding is the foundation for all qualitative data analysis. Therefore, the chosen analysis approach should align with the research goal and data type. Thus, it is essential to determine the analytical approach early on, ensuring it is aligned with the research question and study design.

### 4.4 Procedures, and some points for consideration

The steps of qualitative data analysis are organizing and becoming familiar with the data, followed by pattern recognition and creating themes. The successive steps are coding, category construction, and theme building. Codes are words or phrases that symbolize a single concept, and categories are a collection of such codes. Themes, however, are more about larger or overarching concepts. For example, based on my own engagement with the following data from my personal diary:

*In the 21*^*st*^ *century, self-directed and motivated learning is crucial. However, there never seems to be enough time in the day. Often, I find myself too exhausted to focus on reading. Day-to-day work and professional development present more pressing issues. Time constraints from work and professional growth are significant. At home, I want to engage in other activities, but fatigue from the office limits my productivity. Numerous pressing matters, such as virtual meetings, overly ambitious faculty engagements, and assisting students with their learning, further strain my time. Consequently, time is a constraint in both my personal and professional life. The primary challenge lies in balancing work and family responsibilities*.

(Source: Personal Diary, December 2023)

From the codes to categories to the theme of the data as illustrated in Table 2.

However, the fact that “quotes,” “codes,” “categories,” and “themes” are an iterative process in qualitative research lends itself to a cyclical pattern where I actively seek a deeper appreciation. First, direct quotations from personal data are collected and studied. These quotations are then coded in higher abstraction; the essential meaning unit is abstracted. And as more and more data comes in, these codes get combined into more general ones that illuminate similar patterns. Lastly, the categories are aggregated into overall themes that allow the research problem to be better understood. This step-by-step approach makes it possible to continuously refine and gain detailed insights while analyzing subsequent data. I have also utilized peer debriefing and member checking activities with colleagues of mine working at Kathmandu University School to secure the credibility of quoting, coding, categorizing, and thematizing the data I provided on the basis of the example. Similarly, the number of codes employed is contingent on the question(s) being explored. In general, one starts with a large number of codes and refines through iterative analysis and peer debriefing, as I demonstrated in Table 2. Typically, 25–30 codes generate 4–5 themes, and a single transcript usually yields quotations from 10 to 15 codes.

### 4.5 Achieving saturations: when enough is enough

Data saturation refers to the point at which the collection of additional data no longer adds new information or themes (Rahimi and Khatooni, 2024; Saunders et al., 2018; Yang et al., 2022), so that all data are thoroughly and credibly analyzed. Critical factors to achieve saturation are theoretical saturation, in which no new theoretical properties are generated, existing categories are fully developed, and cues for saturation, e.g., repetition trends and redundancies in the data. Fresh information constantly fits with the old and never builds new elements. Although the number of participants in a qualitative study may be a consideration (Boddy, 2016; Dahal et al., 2024), it needs to be large enough to be able to reflect the diversity and complexity of the phenomenon under study. The research question, study design, and data saturation influence saturation. Data saturation is one of the major aims of qualitative research (Saunders et al., 2018) and shows that data collection has been completed when no new information or themes are gathered (Rahimi and Khatooni, 2024). Ongoing processes of collecting and analyzing data is a logical cut-off point for determining when new data is no longer providing new insights. Thus, redundancy in the data should be tracked; when much similar information is heard and no new themes are observed, the data may reach saturation (Aldiabat and Le Navenec, 2018). For example, in grounded theory, ‘theoretical saturation' is reached when further data collection ceases to provide new theoretical insights or categories, a concept that is also applicable to other qualitative traditions. In a qualitative study, there is no need for a large sample size, but in order to be representative and distinctive, the sample must be chosen on the basis of the criteria (Dahal et al., 2024). Therefore, in my opinion, the researcher should be familiar with coding saturation and meaning saturation. I cannot explore meaning saturation as it is a concept that relies on the depth and quality of the information to be gathered rather than on the quantity of information. Interacting with peers or colleagues in peer debriefing can be useful to confirm that saturation has been attained (Hennink et al., 2017). Thus, it is beneficial to maintain good records of the collection of data, including when and how it was collected and analyzed, to improve transparency and trustworthiness of the research process (Sebele-Mpofu and Serpa, 2020). Flexibility is also required because the saturation point may not be obvious and potentially depends on the complexity of the research topic and the methods involved. Therefore, attaining saturation ensures that there are enough research findings and/or results available on which to base meaningful conclusions and reflect enough findings and/or results.

### 4.6 Challenges and strategies

As an autoethnographer, I realized that qualitative research often faces several challenges, such as data overload, where diverse data forms, including text, audio, video, and images. These data are collected through interviews, focus group discussions, audio recordings, music and video recordings, journal writing (structured text such as writings, news articles, survey comments, stories, etc.), and unstructured text (conversations, interviews, focus group, transcriptions, etc.) can be overwhelming to manage and analyze (Malterud, 2001). One of the significant threats in today's world for researchers is the heavy reliance on generative artificial intelligence (GenAI) in qualitative studies, which limits the cognitive and evaluative abilities for thinking and writing qualitatively and creatively (Chiu, 2023; Dahal, 2024). Moreover, because qualitative analysis is interpretive by its very nature, it can be subjective, which may result in researcher bias and influence the credibility of findings. Saturation, in which no additional information or themes are found, may be unclear and different between studies and research designs. Furthermore, the process of iterative data extraction, quoting, coding, categorizing, thematizing, and interpretation is both time-intensive and demanding. Ensuring the research is credible, dependable, transferable, and confirmable can also be difficult to do in an adequate manner, thus maintaining rigor (Dahal, 2024). Researchers can use data management programs such as MAXQDA, NVivo, or Atlas.ti to deal with these challenges for handling big data in a convenient way. Likewise, to increase credibility and reduce bias, researchers use triangulation, where they use a variety of data sources or research methods. As such, clear reporting is essential and should not come at the expense of the methodological rigor of the research process (Dahal et al., 2024), and the coding should be documented, as well as the decision and reason for saturation. One way to set up such challenges in the data analysis is to have regular debriefing moments with colleagues or advisors. This preemptive data sharing can contribute to designing data gathering and initial issue scoping. The importance of developing reflexivity, which involves regularly examining one's biases and how they may impact the research process, is also fundamental. Lastly, member checking, or the validation of findings by returning them to the participants to hear if the findings ring true with their truth claims, adds to the credibility and transferability of the study. Therefore, by effectively overcoming these challenges, researchers will be able to improve the quality and reliability of their qualitative studies, and the results and/or outcomes of the qualitative data will likely be strengthened and enlightened.

## 5 Final remarks

Qualitative data analysis is a dynamic and cyclical process, necessitating attention to detail while allowing for both refinement and creativity in using a constructivist approach toward methodological rigor (Dahal et al., 2024; Suter, 2012). Transitioning from getting to know the data and coding it to working on themes and having the information finally be saturated is a means to guarantee meaningful and comprehensive findings and/or results. Hence, by embracing the complexities of qualitative research, researchers can explore in-depth insights that enhance our understanding of human experiences and the social world. As a result, in qualitative research, one moves from the data to saturation in the analysis. The journey starts with early procedures, such as immersion in the data, first coding, and memo writing, which form the framework for future analyses. During the research process, themes are created and refined to extract deeper meanings and patterns from the data. Data saturation is important because it indicates when data collection has been conducted to the extent that nothing new is being learned and no new themes are emerging. This procedure guarantees the quality and reliability of the research results/outputs. Every step presents challenges, such as managing a vast data matrix, adhering to strict rigor standards, and attempting to reduce individual interpretation. Triangulation, reflexivity, and data management tools are some methods that can overcome these challenges and improve the reliability and validity of research. Thus, qualitative data attempts to present thick, bold, and valid images that lend understanding to the desires under investigation and/or the inquiry. Therefore, by wisely pursuing the steps from inception to saturation, researchers can have confidence that their findings/results are novel and meaningful, contributing new knowledge. Table 3 illustrates a concise summary table capturing the core elements of qualitative data analysis (QDA), such as the interpretative process grounded in constructivism, methodological rigor, and creative engagement with data.

**Table 3:** Qualitative data analysis procedures.

**Aspect**	**Key elements**	**Details/description**
Philosophy	Constructivist approach	Knowledge is co-constructed; reality is multiple. Researcher subjectivity is integral to meaning-making.
QDA process	Iterative & dynamic	Non-linear cycle: immersion → coding → categorization → theme development → saturation.
Key procedures	1. Immersion 2. Coding 3. Categorization 4. Theme development	Read/reread data. Label data segments (inductive/deductive). Group codes. Synthesize categories into overarching themes.
Common methods	Autoethnography, PAR, Narrative Inquiry, Grounded Theory, Phenomenology, Ethnography, Case Study	Each method dictates unique analytical lenses and data types (e.g., personal journals, stories, observations).
Analytical techniques	Deductive, Inductive, Content, Thematic, Narrative, Discourse, Phenomenological Analysis	Align technique with research goals (e.g., theory-testing vs. pattern discovery).
Rigor strategies	1. Reflexivity 2. Triangulation 3. Peer debriefing 4. Member checking	Critically examine researcher bias. Cross-validate with multiple data sources/methods. Seek external feedback on themes. Verify interpretations with participants.
Saturation	Endpoint of analysis	No new insights/themes emerge from the data. Indicates thematic sufficiency.
Outcome	Meaningful interpretation	Extracts patterns, themes, and perspectives to understand human experiences and social processes.
Challenges	Data overload, researcher bias, subjectivity, defining saturation, and time intensity	Managed via software (NVivo/ATLAS.ti), transparency, iterative refinement, and collaboration.

## Data Availability

The raw data supporting the conclusions of this article will be made available by the authors, without undue reservation.
